# Improvement in Fatigue during Natalizumab Treatment is Linked to Improvement in Depression and Day-Time Sleepiness

**DOI:** 10.3389/fneur.2015.00018

**Published:** 2015-02-23

**Authors:** Iris-Katharina Penner, Eva Catharina Sivertsdotter, Elisabeth G. Celius, Siegrid Fuchs, Karen Schreiber, Sara Berkö, Anders Svenningsson, for the TYNERGY trial investigators

**Affiliations:** ^1^Department of Cognitive Psychology and Methodology, University of Basel, Basel, Switzerland; ^2^BiogenIdec Sweden AB, Upplands Väsby, Sweden (formerly employed); ^3^Department of Neurology, Oslo University Hospital, Ullevål, Norway; ^4^Department of Neurology, Medical University of Graz, Graz, Austria; ^5^Department of Neurology, Copenhagen University Hospital Rigshospitalet, Copenhagen, Denmark; ^6^BiogenIdec Sweden AB, Upplands Väsby, Sweden; ^7^Department of Pharmacology and Clinical Neuroscience, University Hospital of Northern Sweden, Umeå University, Umeå, Sweden

**Keywords:** fatigue, multiple sclerosis, treatment response, depression, sleepiness

## Abstract

**Background:** Fatigue is a frequent symptom in multiple sclerosis (MS) and often interrelated with depression and sleep disorders making symptomatic treatment decisions difficult. In the single-arm, observational phase IV TYNERGY study, relapsing–remitting MS patients showed a clinically meaningful decrease in fatigue over 1 year of treatment with natalizumab.

**Objective:** To evaluate whether fatigue improvement might be directly linked to improved depression and day-time sleepiness.

**Methods:** Patients were assessed regarding fatigue, depression, and day-time sleepiness. The relation between changes of the two latter symptoms and changes in fatigue was analyzed.

**Results:** After 1 year of natalizumab treatment, the majority of patients (>92%) remained stable or improved in total, motor, and cognitive fatigue. Proportion of patients without depression increased by 17% while proportions of mildly depressed patients or patients with potential major depression decreased by 5 and 12%, respectively. Proportion of patients classified as not being sleepy increased by 13% while proportions of sleepy and very sleepy patients decreased by 11 and 2%, respectively. Most importantly, improved depression and sleepiness were significantly related to improved fatigue.

**Conclusion:** Our findings highlight the importance of patient-reported outcomes in identifying potential benefits of drug treatment beyond its well-established effects on disease activity and disability progression.

## Introduction

Fatigue is defined as an extreme form of exhaustion with obvious negative effects on quality of life. With a prevalence ranging between 53 and 95% (1, 2), it is the most frequent “hidden symptom” in multiple sclerosis (MS). Often, fatigue symptoms force individuals to substantially reduce their workload or to even quit their occupation completely. There is evidence that MS-fatigue is strongly related to depression and sleep disorders (3, 4) although the underlying pathophysiological processes are still not completely understood. From the patient’s perspective, these factors are of particular importance in the context of the overall burden of the disease. However, effective symptomatic treatment specifically for fatigue is still missing, leaving the patient with feelings of helplessness, and the physician unsatisfied.

Patients suffering from obvious fatigue symptoms are often treated with antidepressants, most likely efficacious, partly due to the strong association between depression and fatigue. Further, modafinil, amantadine, and aminopyridine are known as treatment options although the therapeutic efficacy is still a matter of debate.

In terms of MS disease-modifying drugs (DMTs), there are no conclusive data available regarding their efficacy on fatigue symptoms. Studies using first generation DMTs, e.g., interferon (IFN) and glatiramer acetate (GA) have yielded divergent results (5–8) while two recent publications on the impact of natalizumab on fatigue (9, 10) showed significant improvement of symptoms after a 1- and 2-year follow-up period, respectively.

In the prospective, multicentre, open-label, observational phase IV TYNERGY study, patients with relapsing-remitting MS (RRMS) who were naïve to natalizumab treatment at baseline experienced improvement in MS-related fatigue (primary efficacy endpoint) over 1 year of treatment (10). The present data analysis was focused especially on the question whether the amount of fatigue improvement was directly linked to improvements in depression and day-time sleepiness.

## Materials and Methods

### Participants

Eligible patients were prescribed natalizumab according to national guidelines, were 18–65 years old (inclusive) at screening, and presented with at least mild fatigue {as determined by the Fatigue Scale for Motor and Cognitive Functions [FSMC (11)] sum score of ≥43; see Table 1}. Patients who had no symptoms of fatigue (i.e., had an FSMC total score <43 at baseline), had an Expanded Disability Status Scale (EDSS) score ≥6.0, were receiving amphetamine medication, or had major depression (assessed by clinical interview of the patient and review of the medical records) were excluded from the study.

**Table 1:** **FSMC cut-off values**.

Score/subscore	Cut-off value	Grading of fatigue
FSMC sum (total) score	≥43	Mild fatigue
	≥53	Moderate fatigue
	≥63	Severe fatigue
FSMC cognitive score	≥22	Mild cognitive fatigue
	≥28	Moderate cognitive fatigue
	≥34	Severe cognitive fatigue
FSMC motor score	≥22	Mild motor fatigue
	≥27	Moderate motor fatigue
	≥32	Severe motor fatigue

The intent-to-treat population (ITT) included all enrolled patients (*N* = 195). A total of 31 withdrawals occurred over the trial period, leaving 164 patients who completed the trial. More than two-thirds of the 195 patients were female (71.3%) (Table 2). At baseline, the average age was 39.7 years, and the average duration of MS was 8.8 years. The median EDSS score at baseline was 3.0, and two-thirds of the patients experienced a relapse within 6 months prior to the baseline visit. Most patients (86%) had previously received disease-modifying therapy; a third of the patients (31%) had received interferon (IFN) beta in the month prior to inclusion in TYNERGY.

**Table 2:** **Demographics and baseline characteristics**.

Variable	Total ITT population (*N* = 195)
Gender, female, *n* (%)	139 (71.3)
Race, white, *n* (%)	188 (96.4)
Age (years)
Mean (SD)	39.7 (9.2)
Median	39.9
Min, Max	18.3, 63.8
EDSS score, median (range)	3.0 (0.0–7.0)
Received IFN beta therapy in month prior to TYNERGY, *n* (%)	61 (31.3)
Duration of MS (years)
Mean (SD)	8.8 (7.0)
Median	6.7
Min, Max	0.2, 30.5

*SD, standard deviation*.

The study was conducted in compliance with Good Clinical Practices (GCP) and the Declaration of Helsinki, and was approved by the institutional ethical review board at the University Hospital of Northern Sweden, Umeå. Consecutive patients prescribed natalizumab at the participating centers gave their written, informed consent to enter the study after the therapy decision was made.

### Study design

The TYNERGY study used a one-armed trial design to primarily evaluate the change in fatigue after 1 year of natalizumab treatment with a well-defined and validated instrument, the FSMC, designed for use in MS patients. Cut-off values for the clinical categories mild, moderate, and severe MS-related fatigue are shown in Table 1.

Besides fatigue, other important aspects that may have an important effect on functioning and well being of MS patients were assessed at baseline, at month 6, and at month 12. They were: work capacity (assessed by the Capacity for Work Questionnaire – CWQ), health related quality of life (HRQoL), sleepiness, depression, cognitive impairment (assessed by the Symbol Digit Modalities Test – SDMT, and the Paced Auditory Serial Addition Test – PASAT), walking speed, MS disease disability, and overall activity using a step counter that was worn for 7 days the week before the study visit.

The DMTs used prior to initialization of natalizumab were documented. All concomitant medications taken during the trial were recorded and special attention was paid to change in symptomatic fatigue therapy, e.g., modafinil and amantadine. Information on relapses, adverse events (AEs), and serious adverse events (SAEs) were collected.

The first patient’s first visit was on March 23, 2009 and the last patient’s last visit on June 30, 2011. EudraCT number for the Swedish protocol: 2008-008065-35. Clinical Trials.gov identifier: NCT00884481. The study was considered observational in Austria, Norway, and Denmark.

The study was performed at 27 centers: 12 in Sweden, 7 in Norway, 5 in Austria, and 3 in Denmark. Patients were scheduled for five assessment visits (baseline and at months 3, 6, 9, and 12) over a period of 12 months.

Results have been described in detail elsewhere (10).

### Assessment instruments

Fatigue was assessed by the FSMC, a validated 20-item questionnaire specifically developed for MS patients. The FSMC allows separate evaluation of motor and cognitive fatigue and clinical grading of fatigue severity.

Depression was measured by the Center for Epidemiologic Studies Depression Scale (CES-D). The CES-D is a short self-report scale designed to measure depressive symptomatology in the general population. It consists of 20 questions and scores ranging from 0 to 60: a score <15, no depression; A score 15–21, mild-to-moderate depression; A score >21, possibility of major depression.

Day-time sleepiness was assessed by the Epworth Sleepiness Scale (ESS) for day-time sleepiness. The ESS is a short, eight-item questionnaire designed to determine the level of day-time sleepiness. Scores range from 0 to 24: a score in the 0–9 range is considered normal. A score in the 10–24 range indicates that expert medical advice should be sought. A score of ≥10 is considered sleepy. A score of ≥18 is considered very sleepy.

### Statistical analyses

Statistical analyses were based on pooled datasets from all participating countries. All statistical tests were two-sided with a 5% level of significance unless otherwise stated. Fatigue was classified as mild, moderate, or severe, according to the FSMC score cut-off values listed in Table 1. On the basis of FSMC scores at 1 year, patients were categorized into three groups:

- Worsened fatigue (a shift to higher fatigue classifications, e.g., moderate to severe);
- Stable fatigue (no change in fatigue classification);
- Improved fatigue (a shift to lower fatigue classifications, e.g., moderate to mild).

Correlations between changes in FSMC motor and cognitive sub-scores and changes in CES-D and ESS scores were evaluated using Pearson correlation coefficients. Associations between FSMC status (worsened, stable, or improved) and changes in CES-D and ESS at 1 year were assessed by analysis of covariance, with adjustment for baseline scores and antidepressant use.

All statistical analysis and programing were done using SAS v9.2.

## Results

### Changes in fatigue

**Figure 1:**
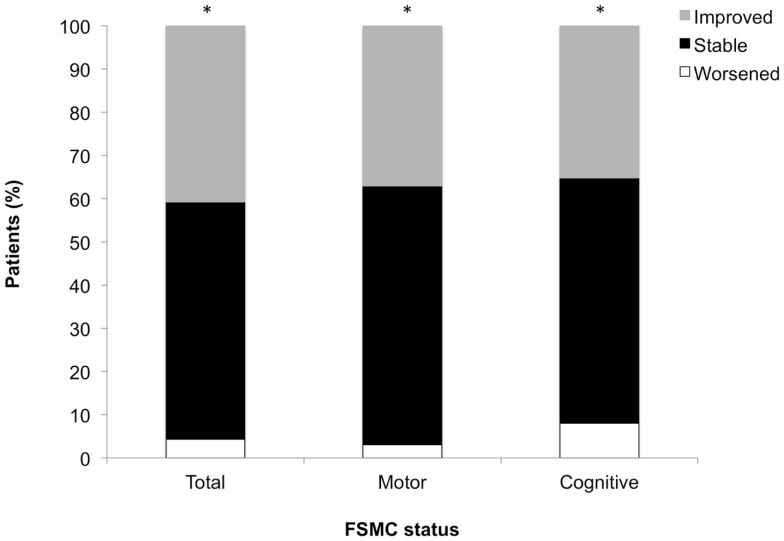
**Changes from baseline in total, motor, and cognitive FSMC scores**. *P < 0.001 across status groups (worsened, stable, improved). Worsened = shift to higher fatigue classification; stable = no change in fatigue classification; improved = shift to lower fatigue classification.

After 1 year of natalizumab treatment, the majority of patients remained stable or improved in FSMC total (96%), motor (97%), and cognitive (92%) scores (10) (see Figure 1).

### Changes in depression and association with changes in fatigue scores

**Figure 2:**
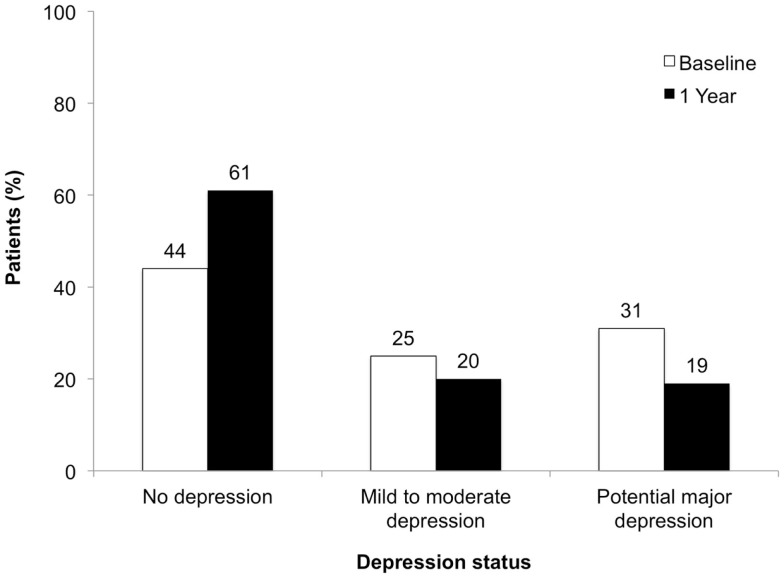
**Depression status at baseline and at 1 year of follow-up**.

**Figure 3:**
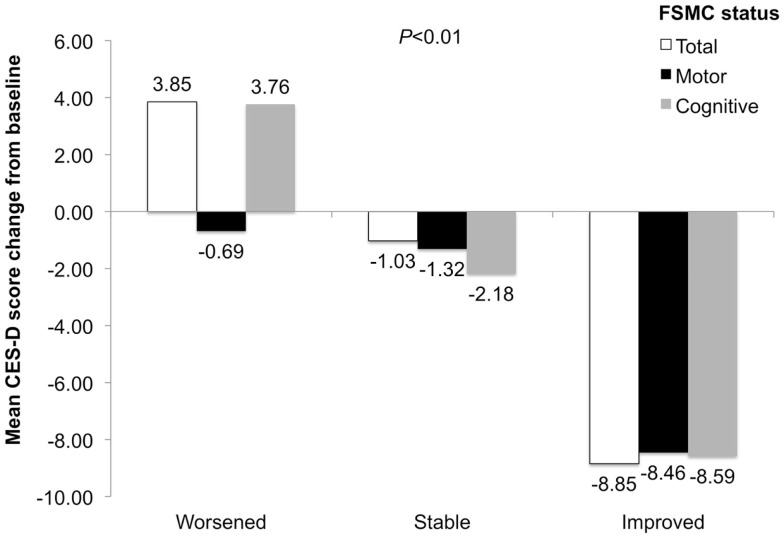
**CES-D score change in patients with worsened, stable, and improved FSMC total, motor, and cognitive scores**. *P* value is for comparison across subgroups of worsened, stable, and improved.

The proportion of patients with no depression increased by 17%, while proportions of patients mildly to moderately depressed or with potential major depression decreased by 5 and 12%, respectively (Figure 2). CES-D score changes differed among worsened, stable, and improved FSMC total/subscale subgroups (*P* < 0.01), with greatest improvements in patients with improved FSMC scores (Figure 3). Improved FSMC total, motor, and cognitive scores were associated with improved mood (correlation coefficients = 0.45, 0.39, 0.47, respectively, *P* < 0.01).

### Changes in sleepiness and association with changes in fatigue scores

**Figure 4:**
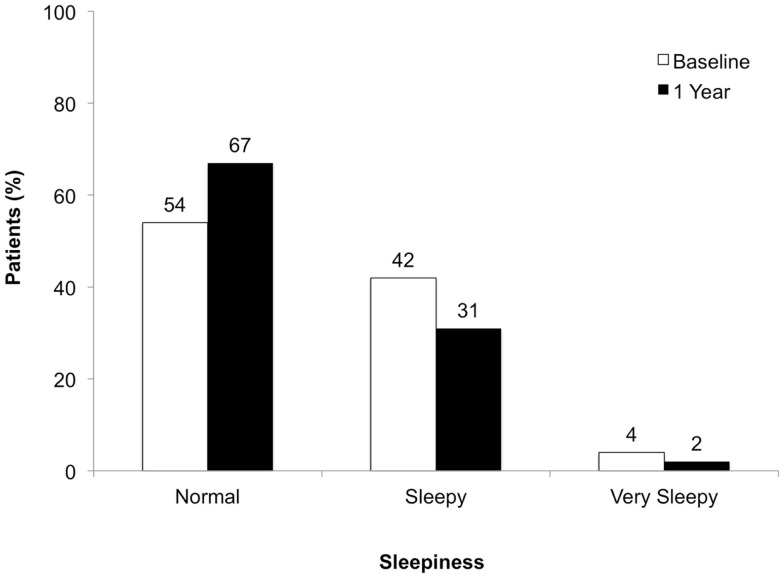
**Degree of sleepiness at baseline and at 1 year of follow-up**.

The proportion of patients classified as not sleepy on ESS increased by 13%. Proportions of patients classified as sleepy or very sleepy decreased by 11 and 2%, respectively (Figure 4).

**Figure 5:**
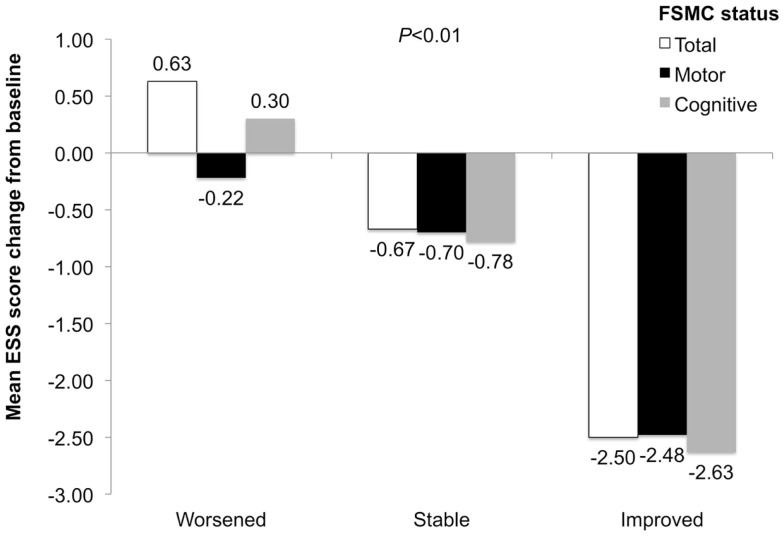
**ESS-score change in patients with worsened, stable, and improved FSMC total, motor, and cognitive scores**. *P* value is for comparison across subgroups of worsened, stable, and improved.

Improvement in FSMC total, motor, and cognitive scores was associated with improved ESS scores (correlation coefficients = 0.44, 0.37, 0.46, respectively, *P* < 0.01). ESS-score changes differed among worsened, stable, and improved total/subscale FSMC subgroups (*P* < 0.01), with greatest improvements in patients with improved FSMC scores (Figure 5).

## Discussion

The present data analysis was focused on the association between already reported clinically meaningful changes in MS-fatigue under natalizumab treatment (10) and strongly associated factors such as depression and day-time sleepiness.

Our results clearly demonstrate that natalizumab-treated MS patients in TYNERGY exhibited not only improvements in fatigue but also in depression, and day-time sleepiness from baseline to year 1. Measures of total, motor, and cognitive fatigue were stable or improved in more than 90% of patients, all of whom had at least mild fatigue at baseline. This large beneficial effect on fatigue symptoms is most likely referable to the high anti-inflammatory efficacy of the drug and the lack of specific side-effects, which are discussed to increase fatigue.

Although patients with major depressive disorder were excluded from the study, 56% of patients had some degree of depression at baseline. After 1 year of natalizumab treatment, the proportion of patients categorized as depressed had decreased to 39%. Mean CES-D scores decreased (improved) by as much as 8.85 points from baseline. This absolute change is greater than the six-point difference between the pre-specified categories of mild-to-moderate depression and possible major depression, suggesting that the absolute score changes observed were clinically meaningful.

The proportion of patients with some degree of sleepiness decreased from 46% at baseline to 33% at year 1. In contrast to the change in depressive symptoms, the absolute changes in ESS scores were smaller than the change of at least eight points that would be needed to shift between categories of sleepiness. The reason for this discrepancy remains unclear. One might assume that the mechanisms underlying sleepiness are more specific than those causing depression. For depression and fatigue, some pathophysiological similarities such as disturbed serotonergic neurotransmission have been reported [e.g., Hanley and Van de Kar (12)]. For Fatigue and sleep disorders, we have evidence of an interrelation (13). The underlying processes, however, are still unknown.

We are aware that an observational trial always runs the risk of influence by a placebo effect, when starting a new and more efficient treatment. However, since all scales displayed highly significant improvements with an additional increase over time, this argument is not likely to be major in explaining our results.

Besides these promising results, we are aware of limitations that need to be addressed. First, the TYNERGY trial lacks of a control group showing that the reported effects are purely driven by the drug. At the time of study start, there was no other second-line treatment available and it was regarded as unethical to include a control arm since all patients had high disease activity. Second, we were not able to study a causal relation among fatigue, depression, and sleepiness but only relations or associations. Nonetheless, it is of clinical importance to realize that improved fatigue symptoms are associated with an increase of patients’ well being in terms of decreased levels of depression and sleepiness. Third, our study was primarily focused on patient-reported outcomes since these tools offer insight into patients’ view and feelings. We did not control for important confounders such as cytokine influence, which is known to be modulated by natalizumab (14) nor did we control for concomitant medication influence. Finally, the fact that more than 30% of our patients received IFNs prior to study inclusion might have driven in part the effects documented under natalizumab treatment. However, when controlling for different previous treatments, the effect of natalizumab on fatigue remained stable speaking in favor of a therapeutic effect.

## Conclusion

Improvement in fatigue, as measured by decreasing total, motor, and cognitive FSMC scores, was associated with improvement of depression status measured by CES-D and improvement of sleepiness status measured by ESS.

While additional research is needed to elucidate a causal relationship among fatigue, depression, and sleepiness in MS, these findings from TYNERGY highlight the important role of patient-reported outcomes in identifying potential benefits of natalizumab treatment beyond its well-established effects on disease activity and disability progression.

## Conflict of Interest Statement

Iris-Katharina Penner: research grants from Bayer Switzerland AG and the Swiss Multiple Sclerosis Society; honoraria for serving as speaker at scientific meetings, as consultant, and as member of scientific advisory boards for Actelion, Bayer Pharma AG, Biogen Idec, Merck Serono, Roche, and Teva Aventis. Elisabeth G. Celius: compensation from Biogen Idec, Merck Serono, Novartis, Sanofi, and Teva for travel to congresses, lectures, and advisory boards. Karen Schreiber: compensation as a consultant for Biogen Idec and Novartis. Siegrid Fuchs: honoraria for lectures and as a member of advisory boards for Bayer Schering, Biogen Idec, Genzyme, Merck Serono, and Novartis. Sara Berkö: employee of Biogen Idec and hold stock in the company. Eva Catharina Sivertsdotter: former employee of Biogen Idec. Anders Svenningsson: research grants from Baxter Medical, Bayer Schering, and Biogen Idec; travel grants and consulting fees from Baxter Medical, Biogen Idec, Merck Serono, and Sanofi-Aventis.
